# Growth patterns of infants with *in- utero* HIV and ARV exposure in Cape Town, South Africa and Lusaka, Zambia

**DOI:** 10.1186/s12889-021-12476-z

**Published:** 2022-01-10

**Authors:** Dorothy C. Nyemba, Emma Kalk, Michael J. Vinikoor, Hlengiwe P. Madlala, Mwangelwa Mubiana-Mbewe, Maureen Mzumara, Carolyn Bolton Moore, Amy L. Slogrove, Andrew Boulle, Mary-Ann Davies, Landon Myer, Kathleen Powis

**Affiliations:** 1 Division of Epidemiology & Biostatistics, Faculty of Health Sciences, School of Public Health and Family Medicine, University of Cape Town, Anzio Road, Observatory, Cape Town, 7925 South Africa; 2 Centre for Infectious Disease Epidemiology and Research, School of Public Health and Family Medicine, University of Cape Town, Cape Town, South Africa; 3 Centre for Infectious Disease Research in Zambia, Lusaka, Zambia; 4 Department of Medicine, University of Alabama at Birmingham, Birmingham, AL USA; 5 Department of Paediatrics & Child Health, Faculty of Medicine & Health Sciences, Stellenbosch University, Worcester, South Africa; 6 Ukwanda Centre for Rural Health, Faculty of Medicine & Health Sciences, Stellenbosch University, Worcester, South Africa; 7 Western Cape Government: Health, Cape Town, South Africa; 8 Department of Internal Medicine and Pediatrics, Massachusetts General Hospital, Boston, MA USA; 9 Department of Immunology and Infectious Diseases, Harvard T.H. Chan School of Public Health, Boston, MA USA

**Keywords:** HIV-exposed uninfected, HIV-unexposed, antiretroviral therapy, weight-for-age, length-for-age, South Africa, Zambia

## Abstract

**Background:**

Infants born HIV-exposed yet remain uninfected (HEU) are at increased risk of poorer growth and health compared to infants born HIV-unexposed (HU). Whether maternal antiretroviral treatment (ART) in pregnancy ameliorates this risk of poorer growth is not well understood. Furthermore, whether risks are similar across high burden HIV settings has not been extensively explored.

**Methods:**

We harmonized data from two prospective observational studies conducted in Cape Town, South Africa, and Lusaka, Zambia, to compare weight-for-age (WAZ), length-for-age (LAZ) and weight-for-length (WLZ) Z-scores between infants who were HEU and HU, converting infant anthropometric measures using World Health Organisation Growth Standards adjusted for age and sex. Linear mixed effects models were fit to identify risk factors for differences in anthropometrics at 6–10 weeks and 6 months by infant HIV exposures status and by timing of exposure to maternal ART, either from conception or later in gestation.

**Results:**

Overall 773 mother-infant pairs were included across two countries: women living with HIV (WLHIV), 51% (*n* = 395) with 65% on ART at conception and 35% initiating treatment in pregnancy. In linear mixed effects models, WAZ and WLZ at 6–10 weeks were lower among infants who were HEU vs HU [β = − 0.29 (95% CI: − 0.46, − 0.12) and [β = − 0.42 (95% CI: − 0.68, − 0.16)] respectively after adjusting for maternal characteristics and infant feeding with a random intercept for country. At 6 months, LAZ was lower [β = − 0.28 CI: − 0.50, − 0.06)] among infants who were HEU, adjusting for the same variables, with no differences in WAZ and WLZ. Within cohort evaluations identified different results with higher LAZ among infants who were HEU from Zambia at 6–10 weeks, [β = + 0.34 CI: + 0.01, + 0.68)] and lower LAZ among infants who were HEU from South Africa [β = − 0.30 CI: − 0.59, − 0.01)] at 6 months, without other anthropometric differences at either site.

**Conclusion:**

Infant growth trajectories differed by country, highlighting the importance of studying contextual influences on outcomes of infants who were HEU.

**Supplementary Information:**

The online version contains supplementary material available at 10.1186/s12889-021-12476-z.

## Introduction

Scale-up of maternal antiretroviral treatment (ART) use in pregnancy to prevent vertical HIV transmission has been one of the most successful global public health programs. As a result, an unprecedented number of women are taking ART at conception and during pregnancy with over 1 million women living with HIV (WLHIV) giving birth annually [1]. This success has dramatically reduced the number of infants who acquire HIV, contributing to a large and growing population of infants with in-utero dual exposure to HIV and antiretroviral (ARV) drugs [2].

While vertical HIV transmission prevention programs have improved the health of women living with HIV and averted infant HIV acquisition, infants who are HIV exposed but uninfected (HEU) experience a higher risk of poor health outcomes compared to infants who are HIV-unexposed (HU). Whether poorer outcomes are due to in-utero exposure to HIV or ARVs, poor maternal health, increased presence of infectious pathogens in households affected by HIV, or poverty related factors often present in households affected by HIV, including food insecurity, has yet to be clearly delineated. Several studies have reported that infants who are HEU experience poorer growth, health and survival outcomes compared to infants who are HU, starting from birth [2–9]. This has brought into question the extent to which fetal exposure to ARVs and the duration of exposure, either from conception or at a later period of gestation, may be contributing to this disparity. Also, infants born HEU have increased risk of infectious morbidity and mortality when compared to infants born HU [10, 11]. While the causes of this increased morbidity in infants who are HEU are multifactorial, in-utero exposure to ARVs may be a contributing factor. Recent studies have shown reassuring results that breastfeeding while on ART is the optimal feeding strategy for HIV-exposed infants in most resource-limited settings [5, 12, 13]. However, it is cause for concern that the comparative growth of infants who are HEU and HU has highlighted early onset of obesity in late infancy [5]. As the HIV epidemic has matured, with programs to prevent vertical transmission of HIV through universal ART for pregnant women, (World Health Organization Option B) and continuation of this ART for life (World Health Organization Option B+), the types of ART recommended for use in pregnancy, as well as the proportion of women on ART prior to conception have evolved. Given few randomized studies evaluating the safety of different ART regimens in pregnancy, and the absence of equipoise to conduct such trials now, ongoing drug safety surveillance is critical. Identifying the safest ART regimens that optimize maternal and child outcomes represents a key public health challenge. Locations with generalized HIV epidemics and high disease burden are best positioned to provide answers.

South Africa’s antenatal HIV prevalence is one of the highest globally, reported as approximately 30% in 2017 [14]. Zambia’s antenatal HIV prevalence has been reported as 13.3% nationally but as high as 30% in Lusaka [15]. In these high prevalence settings, more than 95% of pregnant WLHIV receive ART in pregnancy, resulting in the majority of infants who are HEU having dual exposure to HIV and ARVs. While not all studies show the same early adverse outcomes of in-utero exposures, some have found alterations in growth [8, 9, 16–20]. In particular, infants who are HEU may experience a higher risk of suboptimal growth in infancy [4, 5, 8, 13, 16–18, 20–22] with these effects persisting through to school-going age [7, 23]. Many studies however, (1) lack comparison with children who are HU. This limits the ability to differentiate between the effects of fetal HIV and ARV exposure from effects of the socio-economic environment and health interventions, precluding an understanding of the extent to which biological, socio-economic, or structural factors might be contributing to poor growth among infants who are HEU. (2) Additionally, much of the published research in this area reflects outcomes during the early period of implementation of WHO Option B+ (lifelong triple ART for pregnant and breastfeeding women). As such, there are fewer data on infant growth following ARV exposure from conception and on health outcomes of infants who are HEU in the context of widespread breastfeeding up to 24 months. (3) Furthermore, some of the studies reporting absence of growth differences between infants who are HEU, and HU may not have been adequately powered to detect small but clinically important differences [5, 24, 25]. Pooling of data between studies has not been routinely performed to achieve adequate statistical power to detect these potentially small but clinically meaningful differences. To evaluate the effect of ARV exposure specifically, timing and duration of fetal exposure must be studied among children born to WLHIV. We pooled prospectively collected data from the B-Positive cohort conducted in Cape Town, South Africa, and the B + Readiness cohort in Lusaka, Zambia to evaluate associations between in-utero exposure to HIV/ maternal ART and infant anthropometrics by HIV and maternal ART exposure status, controlling for socio-economic differences.

## Methods

### Study setting

We collated data from two observational prospective studies enrolling pregnant women attending antenatal care (ANC) at primary maternity care facilities in Cape Town, South Africa and Lusaka, Zambia. Mother-infant pairs were followed from the child’s birth to at least 6 months of life. The study in Cape Town (B-Positive study) was conducted at a large primary healthcare facility in Gugulethu, an urban township in Cape Town. The facility serves a population of about 350,000 with an estimated antenatal HIV prevalence of 30% in 2015 [26]. The study in Lusaka (B+ Readiness) was a conducted by the Centre of Infectious Disease Research Zambia (CIDRZ) in Lusaka. The antenatal HIV prevalence in Lusaka was estimated to be 30% in 2014 [15]. Both studies were prospective observational studies.

### Study design and study participants

Consecutive pregnant women ≥18 years of age were recruited at their first ANC visit, at both study sites, regardless of HIV status. Study enrolment in both sites occurred between January 2017 and October 2018. In both sites, women were eligible for this study if they planned to reside in the area with their infants until infants were 6 months old and had a known maternal HIV status. Maternal HIV-positivity was based on clinic records and HIV-negative was based on a negative HIV-1/2 rapid antibody test at cohort enrolment.

### Study procedures

All eligible pregnant women who provided informed written consent were enrolled. Birth anthropometrics were abstracted from the child Road to Health Booklets (RTHB) in South Africa and the Under-5 health booklet in Zambia. Birth weight of newborns was measured within 24 h of birth by health facility nurses. Mother-infant pairs were evaluated by the study teams postnatally between 6 and 10 weeks and at 6 months of life. To be included in this secondary analysis, a woman had to deliver a liveborn, singleton infant, birth weight and/or length data had to be available in the child’s RTHB/Under-5 booklet and have infant anthropometric measurements recorded at either 6–10 weeks and 6 months of life.

### Data collection

Data collected included maternal demographics, pregnancy history, healthcare information, delivery/birth details, child health encounters and feeding practices. Identical standardized questionnaires were administered to all women by trained study interviewers at both study sites. Pregnant women with a negative HIV test at enrolment based on routine rapid antibody test were retested immediately after delivery or during the newborn visit occurring within 3–7 days after delivery, and approximately every 3 months during breastfeeding, as per South African [27] and Zambian standard guidelines [28]. HIV DNA PCR testing of infants occurred at birth, and 6 to 10 weeks in South Africa and at 6 weeks and 6 months in Zambia, per national guidelines. Maternal HIV history and other medical conditions were based on medical records. Per study protocol, during the 6–10 week and 6 month study visits infants were weighed using a calibrated digital infant scale by trained study staff after removal of clothing and diapers. All infant length measurements were taken while the infant was recumbent using an infant length measuring board. Two measurements of infant weight and recumbent length were taken at each visit by study staff and the average was calculated. The study clinician conducted regular anthropometric training for all study staff with structured, supervised and competency assessments. Research Electronic Data Capture (REDCap), a secure, web-based application designed to support data capture for research studies, was employed at both study sites to capture study data and University of Cape Town served as the data centre for the pooled, harmonized data [29]. Sample size calculations were done for independent cohort studies to provide good statistical power to examine associations with maternal and infant factors likely to affect HEU infant growth, adequately adjusting for multiple confounders. We had a unique opportunity to use data from the two studies, allowing for sufficient power to identify any difference in the growth of infants who were HEU compared to HU.

### Exposures and Outcomes

For this analysis, we used data collected during the 6–10 week and 6 month study visits. We evaluated two primary exposures of interest. The first was fetal exposure to HIV, while the second focused on timing of ARV exposure and only included infants who were HEU. A dichotomous variable of either ARV exposure from conception versus later in gestation was derived from maternal ART data. The outcomes of interest were infant weight-for-age (WAZ), length-for-age (LAZ) and weight-for-length (WLZ) z-scores, converted from measured weight and length using the WHO Growth Standards, which adjust for infant age in days and infant sex [30]. Child age at each time point was derived using the visit date and the child’s date of birth. As per WHO guidelines, z-scores out of the range of − 3 and + 3 from the median for the reference population were reviewed and corrected in the event of data capturing errors. Unexplained z-scores out of the range of − 5 and + 5 from the median for the reference population were excluded from the analysis [∼2% observations were omitted in the analysis (*n* = 16)]. We used the WHO guidelines to define underweight as WAZ of less than − 2 standard deviations and stunting as LAZ of less than − 2 standard deviations from the median reference population [30].

### Statistical analysis

Data were analysed using Stata 14.0 (Stata Corporation, College Station, TX, USA) [31]. Maternal and infant characteristics were compared using Wilcoxon rank-sum test and χ^2^ test as appropriate. We presented comparison of maternal and infant characteristics stratified by country in supplementary material. Proportions of secondary outcomes, specifically infants underweight or stunted,

were compared by an infant’s in-utero HIV exposure status and, only among infants who were HEU, by timing of in-utero ARV exposure, from conception vs later in gestation. Univariable and multivariable linear mixed models were fit to compare the primary outcomes, mean WAZ, LAZ and WLZ scores first by in-utero HIV exposure status, then for infants who were HEU, by timing of in-utero ARV exposure. The linear mixed effects models were fitted separately for WAZ, LAZ and WLZ including random effects for intercept and an unstructured correlated covariance matrix was assumed for random effects. All covariates in univariable analyses with a *p*-value of 0.10 were included in multivariable analyses. Additionally, an a priori decision was made to include maternal age in the multivariable model, regardless of univariable p-value [3, 32, 33]. Infant feeding practice was defined as exclusive breastfeeding, formula feeding, or mixed feeding based on maternal report. We performed a sensitivity analysis by restricting our analysis to infants who had similar feeding patterns, that is infants who had exclusive breastfeeding up to 6 months of life.

### Ethical considerations

This study was approved by the University of Cape Town’s Faculty of Health Sciences Research Ethics Committee (UCT-HREC-514/2015 and 749/2015), the Western Cape Government Department of Provincial Health Research Committee (WC-2016RP6_286) and University of Zambia Biomedical Research Ethics Committee (UNZA-REC – 007-12-17). All women participating in the study provided informed written consent for their own participation and that of their child’s.

## Results

**Fig. 1:**
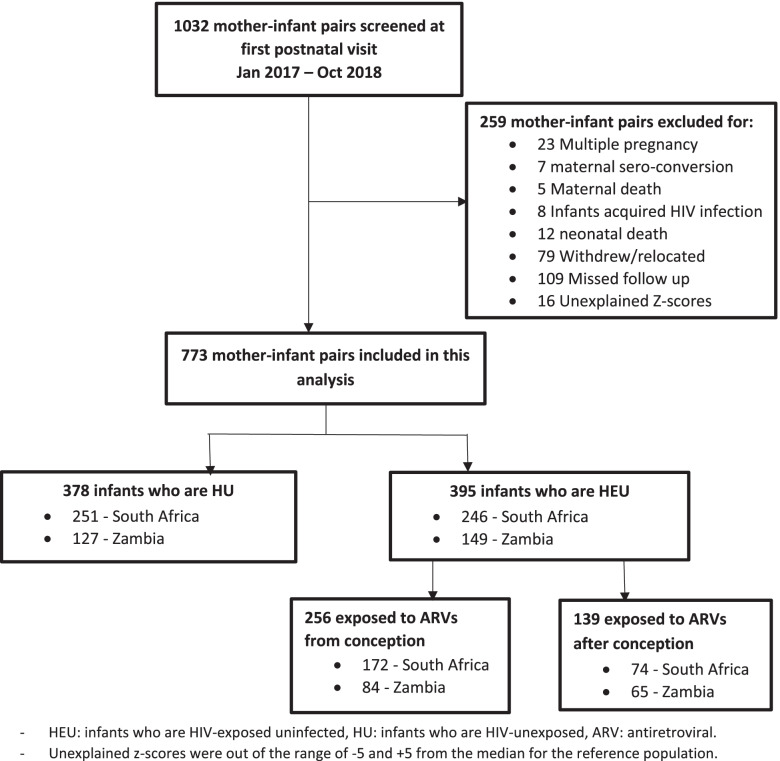
Study flow diagram of participants

A total of 1032 pregnant women were enrolled in the B Positive cohort in Cape Town and the B+ Readiness cohort in Lusaka, 773 mother-infant pairs with live singleton births were included in this analysis. Mother-infant pairs excluded from this analysis have reasons listed in (Fig. 1). Of the 773 infants, 395 (51%) were HEU, while 378 (49%) were HU. Maternal and infant characteristics are shown in Table 1. Women living with HIV were older [31 years; IQR 26–35] than women without HIV, [27 years; IQR 23–32]. Among WLHIV, 65% were on ART at conception and 35% either resumed or initiated ART during pregnancy. Women living with HIV had a slightly but statistically significant lower body mass index (BMI) [27 kg/m^2^; IQR 23–31] and [26 kg/m^2^; IQR 23–32] compared to women without HIV [28 kg/m^2^; IQR 25–34] and [29 kg/m^2^; IQR 24–35] at both 6 to 10 weeks and the 6 month postpartum visits. Overall, fewer WLHIV had completed secondary education. Women living with HIV and women without HIV were similar with respect to employment and marital status. Characteristics of women by country are shown in supplementary material (Table S1). In the Zambia cohort, WLHIV were more likely to have primary education 42% versus 20% in women living without HIV but this was not the same among women from South Africa (7% vs. 3%). In the South Africa cohort, women living without HIV were more likely to be unemployed (71% vs. 61%, *p*-value = 0.03) but there was no difference in employment status by HIV status among women in Zambia cohort (76% vs. 79%, p-value = 0.85) (Table S1). Characteristics of WLHIV by timing of maternal ART initiation is shown in Table 2**.** Women who were already on ART at conception were older ([32 years; IQR 28–36] versus [28 years; IQR 25–33] (*p* < 0.001)) than women who initiated ART during pregnancy and there was no difference all the other demographic characteristics.

**Table 1:** Characteristics of women and infants by HIV status at 6/10 weeks and 6 months in Cape Town, South Africa and Lusaka, Zambia

Maternal characteristics	Total (n, %)	Women living without HIV	Women living with HIV	*P*-value
	773	378 (49)	395 (51)	
**Sociodemographic**				
Age (median, IQR) years	**29 (25–34)**	27 (23–32)	31 (26–35)	< 0.001
Weight at 6/10 weeks (median, IQR) kg	**68 (59–83)**	69 (60–87)	67 (57–80)	0.01
Weight at 6 mons (median, IQR) kg	**69 (58–85)**	71 (60–89)	66 (56–81)	0.001
Height (median, IQR) metres	**1.58 (1.54–1.63)**	1.58 (1.54–1.62)	1.59 (1.55–1.63)	0.03
Maternal BMI at 6/10 weeks (kg/m^2)^	**27 (24–33)**	28 (25–34)	27 (23–31)	< 0.001
Maternal BMI at 6 months (kg/m^2)^	**27 (24–33)**	29 (24–35)	26 (23–32)	< 0.001
**ART initiation**				
Receiving ARVs at conception	256 (65) *	–	256 (65)	
Resuming/initiating ARVs during pregnancy	139 (35) *	–	139 (35)	
ART duration in pregnancy (median, IQR) weeks	**37 (24–39)**	–	37 (24–39)	
**Education level completed**				
Primary	**111 (14)**	33 (9)	78 (20)	< 0.001
Secondary	**636 (82)**	330 (87)	306 (77)	
Tertiary (University)	**26 (4)**	15 (4)	11 (3)	
**Relationship with father of child**				
Married/Cohabiting	**486 (63)**	243 (64)	243 (62)	0.42
Not married/ Non-cohabiting	**287 (37)**	135 (36)	152 (38)	
**Employment status**				
Formal employment	**187 (24)**	81 (21)	106 (27)	0.21
Informal employment	**45 (6)**	26 (6)	22 (6)	
Unemployed/attending school	**541 (70)**	274 (72)	267 (67)	
**Child characteristics**				
**Sex**				
Male	**379 (49)**	189 (50)	190 (48)	0.59
Female	**394 (51)**	189 (50)	205 (52)	
**Age at 6/10 weeks (median, IQR) in days**	**70 (51–73)**	70 (57–74)	69 (49–73)	0.06
**Age at 6 months (median, IQR) in days**	**184 (181–193)**	183 (180–191)	184 (181–196)	0.04
**Weight at birth (median, IQR) kg**				
Male	**3.1 (2.9–3.5)**	3.2 (2.9–3.5)	3.1 (2.8–3.5)	0.24
Female	**3.1 (2.8–3.4)**	3.2 (2.9–3.5)	3.1 (2.8–3.3)	< 0.001
**Low weight at birth (< 2.5 kg)**				
Male	**25 (7)**	15 (8)	10 (5)	0.29
Female	**21 (5)**	7 (4)	14 (7)	0.17
**Weight at 6/10 weeks (median, IQR) kg**				
Male	**5.5 (4.8–6.2)**	5.8 (5.1–6.4)	5.3 (4.7–5.9)	< 0.001
Female	**5.2 (4.6–5.8)**	5.3 (4.8–6.0)	5.0 (4.5–5.7)	0.001
**Weight at 6 months (median, IQR) kg**				
Male	**7.8 (7.1–8.7)**	7.9 (7.2–8.8)	7.6 (7.0–8.6)	0.16
Female	**7.3 (6.7–8.2)**	7.5 (6.8–8.5)	7.2 (6.7–8.0)	0.03
**Length at 6/10 weeks (median, IQR) cm**				
Male	**57 (53–59)**	57 (53–60)	56 (54–58)	0.14
Female	**56 (53–58)**	56 (53–58)	56 (53–58)	0.39
**Length at 6 months (median, IQR) cm**				
Male	**66 (63–68)**	66 (63–69)	65 (63–67)	0.002
Female	**64 (62–67)**	64 (62–67)	64 (62–66)	0.34
**Z-scores at 6/10 weeks, mean (SD)**				
Weight-for-age	**−0.15 (1.17)**	0.05 (1.16)	−0.34 (1.14)	< 0.001
Length-for-age	**−1.07 (1.43)**	−1.02 (1.49)	−1.12 (1.37)	0.35
Weight-for-length	**1.19 (1.78)**	1.43 (1.78)	0.96 (1.76)	< 0.001
**Z-scores at 6 months, mean (SD)**				
Weight-for-age	**0.02 (1.25)**	0.17 (1.24)	−0.10 (1.24)	0.002
Length-for-age	**−0.95 (1.51)**	−0.76 (1.48)	−1.12 (1.53)	0.001
Weight-for-length	**0.95 (1.46)**	0.99 (1.48)	0.92 (1.44)	0.51
**Child feeding practice at 6/10 weeks**				
Exclusive breast feeding	**560 (72)**	287 (76)	273 (69)	< 0.001
Formula feeding only	**138 (18)**	40 (10)	98 (25)	
Mixed breastfeeding and other	**75 (10)**	51 (14)	24 (6)	
**Child feeding practice at 6 months**				
Exclusive breast feeding	**295 (38)**	170 (45)	125 (32)	< 0.001
Formula feeding only	**396 (51)**	150 (40)	246 (62)	
Mixed breastfeeding and other	**82 (11)**	58 (15)	24 (6)	

*IQR* Interquartile range; *n* number of participants* n for WLHIV = 395

**Table 2:** Characteristics of women living with HIV and their infants by timing of maternal ART initiation at 6/10 weeks and 6 months in Cape Town, South Africa and Lusaka, Zambia

Maternal Characteristics	Total (n, %)	Women who start ART during pregnancy	Women on ART at conception	*P*-value
	395	139 (35)	256 (65)	
**Sociodemographic**				
Age (median, IQR) years	**31 (26–35)**	28 (25–33)	32 (28–36)	< 0.001
Weight at 6/10 weeks (median, IQR) kg	**67 (57–80)**	65 (57–76)	68 (57–82)	0.21
Weight at 6 months (median, IQR) kg	**66 (55–81)**	64 (55–78)	68 (58–84)	0.05
Height (median, IQR) metres	**1.59 (1.55–1.63)**	1.58 (1.54–1.62)	1.59 (1.55–1.63)	0.07
Maternal BMI at 6/10 weeks (kg/m2)	**27 (23–31)**	26 (23–31)	27 (23–32)	0.40
Maternal BMI at 6 months (kg/m2)	**26 (23–32)**	25 (23–32)	27 (23–33)	0.12
**ART regimen**				
TDF-XTC-EFV	**318 (80)**	119 (86)	199 (77)	0.08
Other regimens	77 (17) *	20 (14)	57 (20)	
ART duration in pregnancy (median, IQR) weeks	**37 (24–39)**	19 (14–25)	38 (37–39)	
**Education level completed**				
Primary	**78 (20)**	29 (21)	49 (19)	0.34
Secondary	**306 (77)**	104 (75)	202 (79)	
Tertiary (University)	**26 (411 (3))**	6 (4)	5 (2)	
**Relationship with father of child**				
Married/Cohabiting	**243 (62)**	90 (65)	153 (60)	0.33
Not married/ non-cohabiting	**152 (38)**	49 (35)	103 (40)	
**Employment status**				
Formal employment	**106 (27)**	31 (22)	75 (29)	0.23
Informal employment	**22 (6)**	10 (7)	12 (5)	
Unemployed/attending school	**267 (67)**	98 (70)	169 (66)	
**Child characteristics**				
**Child sex**				
Male	**190 (48)**	65 (47)	125 (49)	0.69
Female	**205 (52)**	74 (53)	131 (51)	
**Age at 6/10 weeks (median, IQR) in days**	**69 (49–73)**	68 (47–73)	70 (50–73)	0.23
**Age at 6 months (median, IQR) in weeks**	**26 (26–28)**	26 (26–28)	26 (26–28)	0.15
**Infant weight at birth (median, IQR) kg**				
Male	**3.1 (2.8–3.5)**	3.2 (2.8–3.5)	3.1 (2.9–3.4)	0.44
Female	**3.1 (2.8–3.3)**	3.1 (2.8–3.3)	3.0 (2.8–3.3)	0.64
**Weight at 6/10 weeks (median, IQR) kg**				
Male	**5.3 (4.7–5.9)**	5.2 (4.6–5.6)	5.5 (4.7–6.0)	**0.04**
Female	**5.0 (4.5–5.7)**	5.1 (4.6–5.7)	5.0 (4.4–5.6)	0.54
**Weight at 6 months (median, IQR) kg**				
Male	**7.6 (7.0–8.6)**	7.5 (7.0–8.2)	7.8 (7.1–8.7)	0.11
Female	**7.2 (6.7–8.0)**	7.4 (6.9–8.0)	7.2 (6.6–8.0)	0.25
**Length at 6/10 weeks (median, IQR) cm**				
Male	**56 (54–58)**	55 (53–58)	57 (54–58)	0.28
Female	**56 (53–58)**	55 (53–58)	56 (53–58)	0.63
**Infant length at 6 months (median, IQR) cm**				
Male	**65 (63–67)**	65 (63–67)	65 (63–67)	0.66
Female	**64 (62–66)**	64 (61–66)	64 (62–66)	0.36
**Z-scores at 6/10 weeks, mean (SD)**				
Weight-for-age	**−0.34 (1.14)**	− 0.35 (1.13)	− 0.34 (1.15)	0.92
Length-for-age	**−1.12 (1.37)**	− 1.04 (1.46)	−1.16 (1.32)	0.37
Weight-for-length	**0.96 (1.75)**	0.87 (1.86)	1.01 (1.69)	0.43
**Z-scores at 6 months, mean (SD)**				
Weight-for-age	**−0.10 (1.24)**	− 0.16 (1.19)	− 0.07 (1.27)	0.48
Length-for-age	**−1.12 (1.53)**	−1.26 (1.41)	− 1.04 (1.58)	0.17
Weight-for-length	**0.92 (1.44)**	0.94 (1.39)	0.90 (1.48)	0.78
**Child feeding practice at 6/10 weeks**				
Exclusive breast feeding	**273 (69)**	97 (70)	176 (68)	0.93
Formula feeding only	**98 (25)**	34 (24)	64 (25)	
Mixed breastfeeding and other	**24 (6)**	8 (6)	16 (7)	
**Child feeding practice at 6 months**				
Exclusive breast feeding	**125 (32)**	42 (30)	83 (32)	0.10
Formula feeding only	**246 (62)**	93 (67)	153 (60)	
Mixed breastfeeding and other	**24 (6)**	4 (3)	20 (8)	

*IQR* Interquartile range; *n* number of participants* n for WLHIV = 395

**Fig. 2:**
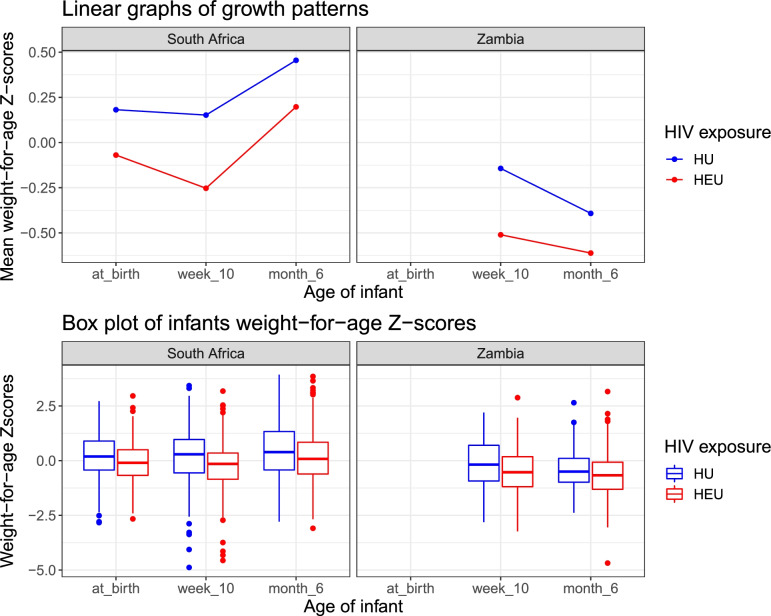
Growth pattern of infants who are HEU and HU from Cape Town, South Africa and Lusaka, Zambia

In the pooled data from South Africa and Zambia, median birthweight for boys was 3.1 kg (IQR 2.9–3.5), with 25 (7%) boys born with low birthweight (< 2500 g). Median birthweight for girls was 3.1 kg (IQR 2.8–3.4), with 21 (5%) girls born with low birthweight. Prevalence of low birthweight did not differ by maternal HIV status: 6% (*n* = 24) of 395 infants who were HEU had low birthweight, compared with 5% (*n* = 22) of 378 infants who were HU (*p* = 0∙88). A significant lower proportion of infants who were HEU exclusively breastfed for a full 6 months compared to infant who were HU (32% versus 45%, *p* < 0.001). Complementary feeding (solid food or nutritive liquids other than breastmilk or formula milk) had been introduced to 6% (*n* = 24) of 395 infants who were HEU versus 14% (*n* = 51) of the 373 infants who were HU by 6 to 10 weeks of age, and 6% (n = 24) of 395 versus 15% (*n* = 58) of 373 by 6 months of age (Table 1). A greater proportion of infants who were HEU switched from exclusive breastfeeding to exclusive formula feeding by 6 months of age (Table 1). Characteristics of infants who are HEU only stratified by timing of maternal ART initiation did not show any difference **(**Table 2**)**. Characteristics of infants stratified by country showed some differences shown in supplementary material (Table S1). At 6 months of life, female infants who were HEU had significantly lower weight compared to female infants who were HU in the South Africa cohort, but this was not the same in the Zambia cohort. In South Africa cohort, infants who were HEU had significantly lower LAZ with mean LAZ − 0.79 versus − 0.45 (*p*-value =0.008) in infants who were HU, but this was not the case in the Zambia cohort (− 0.76 versus − 1.12 and *p*-value = 0.11). At 6 months of life, there was difference in infant feeding practices in South Africa cohort between infants who were HEU compared to HU (p-value < 0.001) but there was no difference in Zambia cohort (*p*-value = 0.23). The growth pattern of infants by country is shown in Fig. 2.

**Fig. 3:**
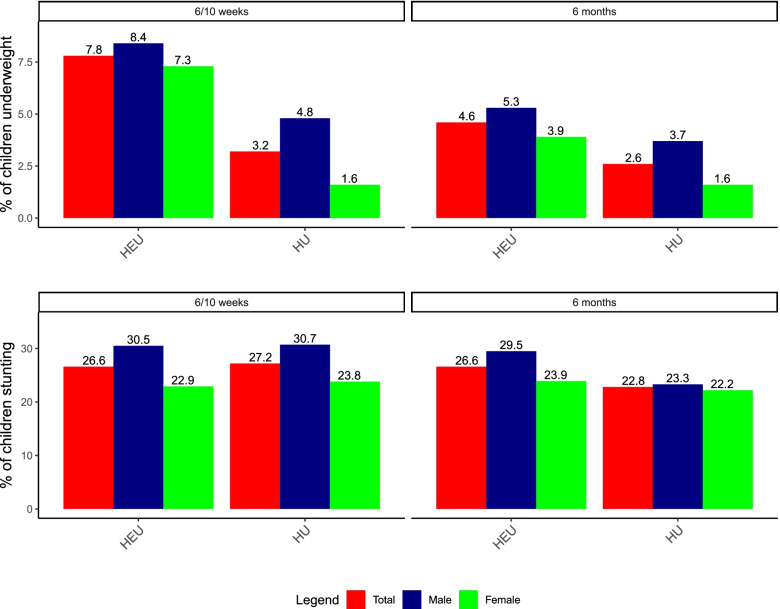
Prevalence of malnutrition by sex, age and HIV exposure among from Cape Town, South Africa and Lusaka, Zambia

In the pooled data between South Africa and Zambia, infants who were HEU were more likely to be underweight at 6 to 10 weeks of life, 7.8% (31 of 395 infants) compared to 3.2% (12 of 378) of infants who were HU (Fig. 3). This difference was less pronounced at 6 months, with 4.6% for infants who were HEU being underweight compared to 2.6% of children who were HU. There was no association between underweight and infant feeding practices at either 6 to 10 weeks or 6 months of age (*p* = 0.39 and *p* = 0.69, respectively).

In linear mixed effects models of pooled results from both cohorts, WAZ and WLZ at 6 to 10 weeks were lower among infants who were HEU compared to those who were HU [β = − 0.29 (95% CI: − 0.46, − 0.12) and β = − 0.42 (95% CI: − 0.68, − 0.15)] after adjusting for maternal age, maternal BMI, infant feeding practice, marital and employment status and country (Table 3). When evaluated by country at 6–10 weeks’ time point, WAZ was lower among infants who were HEU compared to those who were HU at both the South Africa and Zambia country [β = − 0.23 (95% CI: − 0.47, − 0.01) and β = − 0.31 (95% CI: − 0.59, − 0.04)], respectively. At 6–10 weeks, there were no differences for LAZ and WLZ by HIV exposure status in the South Africa cohort, while LAZ was higher [β + 0.34 (95% CI: 0.01, 0.68)] and WLZ was lower [β = − 0.92 (95% CI: − 1.39, − 0.44)] for infants who were HEU compared to those who were HU in the Zambia cohort. At 6 months, LAZ was lower [β = − 0.28 CI: − 0.50, − 0.06)] in the data pooled between the two countries, after adjusting for the same covariates. Evaluation by each cohort individually revealed no differences at 6 months in WAZ and WLZ by group. However, in the South Africa cohort, infants who were HEU had lower LAZ compared to those who were HU in adjusted analyses [β = − 0.30 CI: − 0.59, − 0.01)]. In Zambia cohort, infants who were HEU had lower LAZ but not significantly different compared to those who were HU in adjusted analyses [β = − 0.22 CI: − 0.58, + 0.14)].

**Table 3:** Linear mixed effects models for comparison of WAZ, LAZ and WLZ between infants who were HEU and HU at 6–10 weeks and 6 months

	Anthropometric Measure	Predictor	N	Multivariable at 6–10 weeks		Multivariable at 6 months	
				β (95% CI)	*P*-value	β (95% CI)	*P*-value
*Combined	Weight for age z-score	HU	378	Ref		Ref	
		HEU	395	−0.29 (−0.46 - -0.12)	**0.001**	−0.09 (− 0.27 − + 0.08)	0.31
	Length for age z-score	HU	378	Ref			
		HEU	395	−0.01 (− 0.20 − + 0.21)	0.96	−0.28 (− 0.50 - -0.06)	**0.01**
	Weight for length z-score	HU	378	Ref			
		HEU	395	−0.42 (− 0.68 - -0.16)	**0.002**	+ 0.11 (−0.11 − + 0.33)	0.32
**South Africa**	Weight for age z-score	HU	251	Ref		Ref	
		HEU	246	− 0.23 (−0.47 - -0.01)	**0.04**	−0.11 (− 0.35 − + 0.13)	0.36
	Length for age z-score	HU	251	Ref			
		HEU	246	−0.15 (−0.42 − + 0.11)	0.25	−0.30 (− 0.59 - -0.01)	**0.04**
	Weight for length z-score	HU	251	Ref			
		HEU	246	−0.15 (−0.47 − + 0.16)	0.34	+ 0.10 (− 0.19 − + 0.39)	0.51
**Zambia**	Weight for age z-score	HU	127	Ref		Ref	
		HEU	149	−0.31 (− 0.59 - -0.04)	**0.02**	− 0.09 (− 0.34 - + 0.16)	0.48
	Length for age z-score	HU	127	Ref			
		HEU	149	+ 0.34 (+ 0.01 − + 0.68)	**0.04**	− 0.22 (− 0.58 − + 0.14)	0.23
	Weight for length z-score	HU	127	Ref			
		HEU	149	−0.92 (−1.39 - -0.44)	**< 0.001**	+ 0.08 (− 0.26 − + 0.42)	0.64

Adjusted for maternal age, maternal Body Mass Index (BMI), employment, marital status and infant feeding with random intercept for country

*HEU* Infants who are HIV exposed uninfected; *HU* Infants who are HIV unexposed uninfected; *β* mean change in z-score between HEU and HU; *CI* confidence interval; *N* number of participants

Evaluation of fetal timing of ARV exposure, either from conception or later during gestation did not identify any differences in WAZ, LAZ and WLZ at 6–10 weeks or 6 months among infants who were HEU (Table 4). In a sensitivity analysis, by limiting the analysis to infants who had exclusive breastfeeding up to 6 months, WAZ for pooled data at 6 to 10 weeks was lower among infants who were HEU compared to those who were HU [β = − 0.29 (95% CI: − 0.58 -0.01)] but was resolved 6 months [β = + 0.02 (95% CI: − 0.28 +  0.32)] (Table S2). When evaluated within cohorts, LAZ was significantly higher among infants who were HEU compared to those who were HU in Zambia cohort [β = + 0.61 (95% CI: + 0.09 + 1.13)] while the results from South Africa cohort were in a different direction β = − 0.41 (95% CI: − 0.85 +  0.02)]. All the differences were resolved by 6 months.

**Table 4:** Liner mixed effect models for comparison of WAZ, LAZ and WLZ at 6–10 weeks and 6 months between infants who were HEU with different timing of fetal ARV exposure

	Anthropometric Measure	Predictor - ARV exposure	N	Multivariable at 6–10 weeks		Multivariable at 6 months	
				β (95% CI)	*P*-value	β (95% CI)	*P*-value
*Combined	Weight for age z-score	During pregnancy	139	Ref		Ref	
		From conception	256	+ 0.02 (−0.21 − + 0.26)	0.83	+ 0.04 (− 0.20 − + 0.28)	0.73
	Length for age z-score	During pregnancy	139	Ref			
		From conception	256	−0.24 (− 0.53 − + 0.04)	0.09	+ 0.13 (− 0.18 − + 0.44)	0.41
	Weight for length z-score	During pregnancy	139	Ref			
		From conception	256	+ 0.29 (−0.07 − + 0.66)	0.11	− 0.01 (− 0.32 − + 0.29)	0.92
**South Africa**	Weight for age z-score	During pregnancy	74	Ref		Ref	
		From conception	172	0.08 (−0.24 − + 0.40)	0.61	0.01 (− 0.32 − + 0.35)	0.94
	Length for age z-score	During pregnancy	74	Ref			
		From conception	172	−0.35 (− 0.73 − + 0.03)	0.07	0.03 (− 0.37 − + 0.43)	0.86
	Weight for length z-score	During pregnancy	74	Ref			
		From conception	172	0.53 (0.06–1.01)	**0.02**	0.01 (−0.41 − + 0.42)	0.96
**Zambia**	Weight for age z-score	During pregnancy	65	Ref		Ref	
		From conception	84	−0.11 (−0.48 − + 0.27)	0.58	0.06 (− 0.31 - + 0.43)	0.74
	Length for age z-score	During pregnancy	65	Ref			
		From conception	84	−0.15 (−0.61 − + 0.30)	0.51	0.26 (− 0.25 − + 0.78)	0.31
	Weight for length z-score	During pregnancy	65	Ref			
		From conception	84	−0.04 (−0.61 − + 0.68)	0.90	−0.09 (− 0.71 − + 0.39)	0.71

Adjusted for maternal age, maternal bmi, employment, marital status and infant feeding with random intercept for country

Model limited to infants who are HIV exposed uninfected by timing to exposure to ARVs

*β* mean change in z-score between infants who are HEU and HU; *CI* confidence interval; *N* number of participants

## Discussion

In this prospective cohort of pregnant women seeking ANC at peri- urban public health care facilities in South Africa and Zambia, we found that infants who were HEU experienced lower mean WAZ and WLZ at 6 to 10 weeks of age compared to infants who were HU. However, these differences were no longer present by 6 months of life. In contrast, we found that infants who were HEU experienced lower mean LAZ at 6 months of age compared to those who were HU, despite the fact that LAZ was similar between these two groups at 6 to 10 weeks of life. In evaluating timing of ARV exposure for infants who were HEU, mean WAZ, LAZ and WLZ did not vary between those infants with foetal exposure from time of conception and those exposed later in gestation.

Our finding of lower WAZ at 6 to 10 weeks among infants who were HEU is consistent with several studies in African populations [2, 5, 9, 19]. It was reassuring that HEU appeared to catch-up in terms of WAZ by 6 months. On the other hand, rapid catch-up growth in infancy may have negative consequences, including a future risk of obesity and cardiovascular disease in adulthood [5, 7]. We tested for an interaction between infant feeding practice and country comparing infants who were HEU with HU and observed that mean WAZ and WLZ were not altered by different feeding practices in the two countries. While our study, and other studies have reported lower WAZ among infants who were HEU born to women receiving ART in pregnancy compared to infants born to women without HIV [5, 7, 9, 19, 34], the aetiology of differences in growth from birth and timing of catch-up in growth between infants who are HEU compared to those who are HU requires further investigation. While equipoise no longer exists to conduct a randomized trial of timing of fetal ARV exposure, large, well designed studies that equally evaluate biological, social, and structural aetiologies would advance confidence in identifying the safest ARV regimens for use in pregnancy and may identify modifiable risk factors to ensure that birth anthropometrics and growth is comparable between infants who were HEU and those who are HU.

Pooling of data suggested the absence of significant differences in LAZ at 6 to 10 weeks by infant HIV exposure status. Yet poorer linear growth was observed among infants who were HEU compared to those who are HU, despite the absence of significant differences in WAZ and WLZ at this time point between the two groups. Whether this finding reflects foetal programming following exposure to either HIV or ARVs versus more sustained inadequacy of gestational nutritional needs requires further investigation. In a Brazilian birth cohort [17], it is cause of concern that longer follow-up through 24 months of life demonstrated persistently poorer linear growth among children who were HEU compared to those who were HU. We noted that child growth trajectories differed by country, highlighting the importance of studying contextual influences on outcomes of infants who are HEU.

Although we did not find any difference for WAZ, LAZ and WLZ between infants who were exposed to ARVs from conception compared to those exposed later in gestation, the association between in-utero ARV exposure and adverse growth beyond infancy is not clear [5, 9, 19, 34]. There is still uncertainty as whether an association exists between a specific ARV drug or regimen and poorer growth in infants who are HEU. In our study, the majority of women received an efavirenz (EFV)-containing regimen, precluding robust analysis of differences in infant growth by maternal ARV regimen.

In our cohort, the percentage of infants who were HEU and more likely to be underweight at 6 to 10 weeks of life was higher compared to infants who were HU and this persisted through 6 months of life. However, the proportion of infants who were underweight at 6 months was lower. This may be explained by infant feeding practices, as a higher proportion of infants who were HEU had switched from exclusive breastfeeding to formula feeding by 6 months. It is concerning that breastfeeding durations were short, particularly among infants who were HEU. Our findings are consistent with a study from Botswana [9], Brazil [17] and South Africa [5], where LAZ scores were lower from 6 months of life. This decline in LAZ could be coinciding with early weaning of infants from exclusive breastfeeding because we did not observe the decline when we restricted the analysis to infants who were exclusively breastfeeding by 6 months. This finding of similar growth pattern between infants who were HEU and HU who were exclusively breastfeeding at 6 months is reassuring given the exposure to ARVs in-utero and through the breastfeeding period. However, there is need for further studies to quantify optimal length of breastfeeding for infants who were HEU.

This study had limitations. We could not assess intrauterine growth restriction because of lack of robust measurement for gestational age at birth for both countries. We limited our analysis to mother-infant pairs who had data at all three time points, potentially introducing a survival bias that precluded the detection of growth differences between infants who were HEU and those who were HU. Unmeasured confounding is always a concern with observational research and differences between mothers with and without HIV could not be fully reconciled. However, efforts were made to minimise confounding in our study design and analysis. Additionally, inclusion of a comparator group of women without HIV and their children from the same communities as the WLHIV minimized socio-demographic differences. While we may have had greater power to detect true differences in anthropometrics by HIV-exposure groups, contextual differences between the two countries masked significant differences present at a single country. Maternal education and employment status were differently distributed in women living with HIV compared to women without HIV between the two countries. We also noted differences in feeding practices between infants who were HEU compared to HU at 6 months in South Africa, but this was not the same in Zambia. These contrasting circumstances could have influenced growth patterns in opposite direction for infants who were HEU compared to HU between South Africa and Zambia as observed in our study.

## Conclusion

In our study, infant growth trajectories differed by country, highlighting the importance of studying contextual influences when conducting outcomes research for the population of infants who are HEU. While pooling of data increases the power to detect small but clinically meaningful differences, studies that rely upon pooling data need to find sound approaches to measurement of contextual heterogeneity between sites for exposures directly associated with the outcome or which mediate the outcome of interest. More data and longer periods of evaluation are needed to ensure that infants who were HEU are achieving comparable growth outcomes to infants born to women without HIV.

## Supplementary Information

**Additional file 1.** Supplementary material

**Additional file 2: Table S1.** Characteristics of women and infants by HIV status and site at 6/10 weeks and 6 months in Cape Town, South Africa and Lusaka, Zambia.

**Additional file 3: Table S2.** Linear mixed effects models for comparison of WAZ, LAZ and WLZ at 6-10 weeks and 6 months of life among infants exclusively breastfed for 6 months of life by infant HIV exposure status.

## Data Availability

The datasets used and analysed during this current study are available from corresponding author on request.

## Abbreviations

ANC: Antenatal clinic
ARV: Antiretroviral
ART: Antiretroviral therapy
GA: Gestational age
HEU: HIV-exposed uninfected
HU: HIV-unexposed
LAZ: Length-for-age
WLHIV: Women living with HIV
WAZ: Weight-for-age
WLZ: Weight-for-length
SD: Standard deviation

## References

[1] UNAIDS. AIDSinfo http://aidsinfo.unaids.org/ (accessed May 2, 2019). 2018.

[2] Evans C, Jones CE, Prendergast AJ (2016). HIV-exposed, uninfected infants: new global challenges in the era of paediatric HIV elimination. Lancet Infect Dis.

[3] Chilyabanyama ON, Chilengi R, Laban NM, Chirwa M, Simunyandi M, Hatyoka LM (2021). Comparing growth velocity of HIV exposed and non-exposed infants: An observational study of infants enrolled in a randomized control trial in Zambia. PLoS One.

[4] Ejigu Y, Magnus JH, Sundby J, Magnus MC (2020). Differences in growth of HIV-exposed uninfected infants in Ethiopia according to timing of in-utero antiretroviral therapy exposure. Pediatr Infect Dis J.

[5] le Roux SM, Abrams EJ, Donald KA, Brittain K, Phillips TK, Nguyen KK (2019). Growth trajectories of breastfed HIV-exposed uninfected and HIV-unexposed children under conditions of universal maternal antiretroviral therapy: a prospective study. Lancet Child Adolesc Health.

[6] Le Roux SM, Abrams EJ, Nguyen K, Myer L (2016). Clinical outcomes of HIV-exposed, HIV-uninfected children in sub-Saharan Africa. Trop Med Int Health.

[7] Moseholm E, Helleberg M, Sandholdt H, Katzenstein TL, Storgaard M, Pedersen G (2019). Children Exposed or Unexposed to Human Immunodeficiency Virus: Weight, Height, and Body Mass Index During the First 5 Years of Life—A Danish Nationwide Cohort. Clin Infect Dis.

[8] Neary J, Langat A, Singa B, Kinuthia J, Itindi J, Nyaboe E (2021). Higher prevalence of stunting and poor growth outcomes in HIV-exposed uninfected than HIV-unexposed infants in Kenya. AIDS (London, England).

[9] Powis KM, Smeaton L, Hughes MD, Tumbare EA, Souda S, Jao J (2016). In-utero triple antiretroviral exposure associated with decreased growth among HIV-exposed uninfected infants in Botswana. AIDS (London, England).

[10] Slogrove AL, Johnson LF, Powis KM. Population-level mortality associated withHIV exposure in HIV-uninfected infants in Botswana and South Africa: a model-based evaluation. J Tropical Pediatrics. 2019;65(4):373-9. 10.1093/tropej/fmy064 PMC6703783 30321432

[11] Taron-Brocard C, Le Chenadec J, Faye A, Dollfus C, Goetghebuer T, Gajdos V (2014). Increased risk of serious bacterial infections due to maternal immunosuppression in HIV-exposed uninfected infants in a European country. Clin Infect Dis.

[12] Kapito-Tembo AP, Bauleni A, Wesevich A, Ongubo D, Hosseinipour MC, Dube Q (2021). Growth and Neurodevelopment Outcomes in HIV-, Tenofovir-, and Efavirenz-Exposed Breastfed Infants in the PMTCT Option B+ Program in Malawi. J Acquir Immune Defic Syndr..

[13] Pillay L, Moodley D, Emel LM, Nkwanyana NM, Naidoo K (2021). Growth patterns and clinical outcomes in association with breastfeeding duration in HIV exposed and unexposed infants: a cohort study in KwaZulu Natal, South Africa. BMC Pediatr.

[14] Woldesenbet SA, Kufa T, Barron P, Ayalew K, Cheyip M, Chirombo BC (2020). Assessment of readiness to transition from antenatal HIV surveillance surveys to PMTCT programme data-based HIV surveillance in South Africa: The 2017 Antenatal Sentinel HIV Survey. Int J Infect Dis.

[15] Republic of Zambia NAC. Zambia Country Report - Monitoring the Declaration of Commitment on HIV and AIDS and the Universal Access. https://www.uniaids.org/sites/default/files/country/documents/ZMB narrative report 2015. Accessed 01 October 2020. 2014.

[16] Aizire J, Sikorskii A, Ogwang LW, Kawalazira R, Mutebe A, Familiar-Lopez I (2020). Decreased growth among antiretroviral drug and HIV exposed uninfected versus unexposed children in Malawi and Uganda. AIDS (London, England).

[17] Hofer CB, Keiser O, Zwahlen M, Lustosa CS, CisneFrota AC, de Oliveira RH (2016). In utero exposure to antiretroviral drugs: effect on birth weight and growth among HIV-exposed uninfected children in Brazil. Pediatr Infect Dis J.

[18] Jumare J, Datong P, Osawe S, Okolo F, Mohammed S, Inyang B (2019). Compromised growth among HIV-exposed uninfected compared with unexposed children in Nigeria. Pediatr Infect Dis J.

[19] Ramokolo V, Goga AE, Lombard C, Doherty T, Jackson DJ, Engebretsen IM (2017). In Utero ART Exposure and Birth and Early Growth Outcomes Among HIV-Exposed Uninfected Infants Attending Immunization Services: Results From National PMTCT Surveillance, South Africa. Open Forum. Infect Dis.

[20] Sirajee R, Conroy AL, Namasopo S, Opoka RO, Lavoie S, Forgie S (2021). Growth Faltering and Developmental Delay in HIV-Exposed Uninfected Ugandan Infants: A Prospective Cohort Study. J Acquir Immune Defic Syndr.

[21] Karakochuk CD, Whitfield KC, Rappaport AI, Barr SI, Vercauteren SM, McLean J (2017). Comparison of four immunoassays to measure serum ferritin concentrations and iron deficiency prevalence among non-pregnant Cambodian women and Congolese children. Clin Chem Lab Med.

[22] Sudfeld CR, Lei Q, Chinyanga Y, Tumbare E, Khan N, Dapaah-Siakwan F (2016). Linear growth faltering among HIV-exposed uninfected children. J Acquir Immune Defic Syndr (1999).

[23] Rosala-Hallas A, Bartlett JW, Filteau S (2017). Growth of HIV-exposed uninfected, compared with HIV-unexposed, Zambian children: a longitudinal analysis from infancy to school age. BMC Pediatr.

[24] Lane CE, Bobrow EA, Ndatimana D, Ndayisaba GF, Adair LS (2019). Determinants of growth in HIV-exposed and HIV-uninfected infants in the K abeho Study. Matern Child Nutr.

[25] Msukwa MT, Estill J, Haas AD, van Oosterhout JJ, Tenthani L, Davies M-A (2018). Weight gain of HIV-exposed, uninfected children born before and after introduction of the ‘Option B+‘programme in Malawi. Aids..

[26] Myer L, Phillips T, Manuelli V, McIntyre J, Bekker L-G, Abrams EJ (2015). Evolution of antiretroviral therapy services for HIV-infected pregnant women in Cape Town, South Africa. J Acquir Immune Defic Syndr (1999).

[27] National Department of Health (2017). The 2015 National Antenatal Sentinel HIV & Syphilis Survey, South Africa.

[28] Lusaka, The Government of the Republic of Zambia (2019). ANC guidelines for a positive pregnancy experience. Health Mo.

[29] Paul A, Harris RT, Thielke R, Payne J, Gonzalez N, Conde JG (2009). Research electronic data capture (REDCap) – A metadata-driven methodology and workflow process for providing translational research informatics support. J Biomed Inform.

[30] World Health Organization. WHO Anthro (version 3.2.2, January 2011) and macros. World Health Organization, Geneva, Switzerland http://www.whoint/childgrowth/software/en (accessed Dec 15, 2019). 2011.

[31] StataCorp. (2017). Stata Statistical Software: Release 15.

[32] Nyemba DC, Kalk E, Madlala HP, Malaba TR, Slogrove AL, Davies M-A (2021). Lower birth weight-for-age and length-for-age z-scores in infants with in-utero HIV and ART exposure: a prospective study in Cape Town, South Africa. BMC Pregnancy Childbirth.

[33] Santosa WB, Staines-Urias E, Tshivuila-Matala CO, Norris SA, Hemelaar J (2019). Perinatal outcomes associated with maternal HIV and antiretroviral therapy in pregnancies with accurate gestational age in South Africa. Aids..

[34] Powis KM, Smeaton L, Ogwu A, Lockman S, Dryden-Peterson S, van Widenfelt E (2011). Effects of in utero antiretroviral exposure on longitudinal growth of HIV-exposed uninfected infants in Botswana. J Acquir Immune Defic Syndr (1999).

