# Supplementary material for: Growth patterns of infants with in- utero HIV and ARV exposure in Cape Town, South Africa and Lusaka, Zambia
Source: BMC Public Health. 2022 Jan 10;22:55. doi: 10.1186/s12889-021-12476-z (PMC8744341; doi:10.1186/s12889-021-12476-z)
Supplement: Supplementary file 1 — Additional file 1. Supplementary material [file 12889_2021_12476_MOESM1_ESM.docx]

**Supplementary material**

**Method and procedure of data collection**

We collated data from two observational prospective studies enrolling women in pregnancy from attendees at public sector antenatal clinics (ANCs) in Cape Town, South Africa and Lusaka, Zambia. Both studies enrolled women living with and without HIV and followed mother-infant pairs postnatally. The studies in Cape Town (B-Positive study) in Lusaka (B + Readiness study) recruited women antenatally and followed mother-infant pairs postnatally through from the child’s birth to at least 6 months of life. Study enrolment in both sites occurred between January 2017 and October 2018. In both sites, women were eligible for this study if they planned to reside in the area with their infants and had a confirmed maternal HIV status, either infected or uninfected, at time of study enrolment. For women not known to be living with HIV, a rapid antibody test was used to confirm their HIV status which is standard of care in routine ANC services. In the B-Positive study in Cape Town, gestational age was assessed by ultrasound at enrolment by dedicated study ultra-sonographer and repeated at all subsequent antenatal visits while in the B + Readiness study in Lusaka, gestational age was assessed using the last menstrual period date (LMP). In both sites, standardized questionnaires were administered to all women by trained study interviewers to collect data that included maternal demographics, pregnancy history, healthcare information and after delivery, child healthcare and feeding practices.

In both study sites, birth weight of new-borns was measured within 24 hours of birth by health facility nurses and birth anthropometrics were abstracted from the child Road to Health Booklets (RTHB). Mother-infant pairs were evaluated by the study teams postnatally 10 weeks and 6 months of life in the B-Positive study and 6 weeks and 6 months of life in the B + Readiness study. Pregnant women with a negative HIV test at enrolment based on routine rapid antibody test were retested immediately after delivery or during the new-born visit at 3-7 days after delivery and approximately every 3 months during breastfeeding, as per South African [1] and Zambian standard guidelines [2]. Infants who were HEU had an HIV DNA PCR test at birth and repeated at 6/10 weeks (standard of care in both sites). For the studies, maternal HIV history and other medical conditions were based on medical records. Per study protocol, all infants were weighed using a calibrated digital infant scale by trained study staff after removal of clothing and diapers at the 6-10 week and 6 month postnatal study visits. All baby length measurements were taken using a baby length measuring board. Two measurements of infant weight and length were taken at each visit by study staff and the average calculated.

| **Variable/Procedure** | **B-Positive study, Cape Town** | **B + Readiness study, Lusaka** |
| --- | --- | --- |
| **Gestational age** | Ultra-sound at enrolment and at delivery | LMP at study enrolment and at delivery |
| **Maternal HIV status** | Rapid antibody test | Rapid antibody test |
| **Maternal ART** |  |  |
| TDF-FTC-EFV | 193 (78) | 145 (97) |
| AZT-3TC-NVP | 2 (1) | 0 (0) |
| TDF-3TC-LPV/r | 2 (1) | 0 (0) |
| AZT-3TC-EFV | 1 (1) | 0 (0) |
| Other regimen | 48 (19) | 4 (3) |
| Maternal demographics and medical history | Standardized questionnaires were administered to all women by trained study interviewers | Standardized questionnaires were administered to all women by trained study interviewers |
| **Infant HIV status** | Infants who were HEU had an HIV DNA PCR test at birth and repeated at 10 weeks | Infants who were HEU had an HIV DNA PCR test at birth and repeated at 6 weeks and 6 months |
| **Newborns exposed to maternal HIV infection** | Nevirapine and AZT for 12 weeks or when still on breastfeeding | Nevirapine and AZT for 6 weeks or when still on breastfeeding |
| Visit schedules | At birth | At birth |
|  | 10 weeks | 6 weeks |
|  | 6 months | 6 months |
| Birth anthropometrics | Extracted from the child Road to Health Booklets | Extracted from the child Road to Health Booklets |
| Child healthcare and feeding practices | Standardized questionnaires were administered to all women by trained study interviewers | Standardized questionnaires were administered to all women by trained study interviewers |

**References**

1.National Department of Health. The 2015 National Antenatal Sentinel HIV & Syphilis Survey, South Africa 2017.

2.The Government of the Republic of Zambia. ANC guidelines for a positive pregnancy experience. In: Health Mo, editor Lusaka 2019.
